# Relationship Between Retinopathy of Prematurity and Anemia and Red Blood Cell Transfusions in Very Premature/Very-Low-Birth-Weight Neonates

**DOI:** 10.3390/diagnostics16131967

**Published:** 2026-06-24

**Authors:** Raluca Mihețiu, Anne Claudia Stefanuț, Mădălina Claudia Hapca, Tudor Călinici, Simona-Delia Nicoară

**Affiliations:** 1 Doctoral School, “Iuliu Hațieganu” University of Medicine and Pharmacy, 8, V. Babes Str., 400012 Cluj-Napoca, Romania; 2 Neonatology-Preterm Department, Emergency Pediatric Hospital, Calea Motilor, No 68, 400370 Cluj-Napoca, Romania; 3 Ophthalmology Clinic, Emergency County Hospital, 3–5 Clinicilor Str., 400006 Cluj-Napoca, Romania; claudiastefanut@yahoo.com (A.C.S.); madalina.prodan06@gmail.com (M.C.H.); 4 Department of Ophthalmology, “Iuliu Hațieganu” University of Medicine and Pharmacy, 8, V. Babes Str., 400012 Cluj-Napoca, Romania; 5 Department of Medical Informatics and Biostatistics, “Iuliu Hațieganu” University of Medicine and Pharmacy, L. Pasteur Str. No 6, 400349 Cluj-Napoca, Romania; tcalinici@umfcluj.ro

**Keywords:** retinopathy of prematurity, anemia, RBC transfusion, preterm infants

## Abstract

**Aim:** Retinopathy of prematurity (ROP) is the leading cause of blindness in preterm infants. In this study, we evaluated the potential role of anemia and packed red blood cell (RBC) transfusions as risk factors in ROP development. **Methods:** A retrospective cohort study was conducted on premature infants who met the following inclusion criteria: infants with gestational age (GA) ≤ 32 weeks and very low birth weight (VLBW) who were admitted to the Neonatology-Preterm Department of Emergency Pediatric Hospital Cluj-Napoca during a two-year period (from 1 January 2023 to 31 December 2024). We investigated differences in the following perinatal characteristics between the two groups, those with ROP and those without: GA, birth weight (BW), severe respiratory distress syndrome, early-onset and late-onset sepsis, hemoglobin (Hb) levels, and RBC transfusions. We used the statistically significant variables to perform binary logistic regression. **Results:** A total of 124 newborns were recruited, with the following inclusion criteria: GA ≤ 32 weeks and BW ≤ 1500 g, of whom 79 received at least one RBC transfusion prior to 36 weeks corrected GA. Of them, 48 developed ROP with an incidence of 38.7%. In 20 cases, ROP required treatment. To adjust for clinical illness, a binary logistic regression model was created, including known risk factors for ROP and illness severity (GA, severe respiratory distress syndrome, and early- and late-onset sepsis) that were closely related to the risk of ROP development. For this regression model, Nagelkerke R-squared = 0.358, *p* < 0.001, and the AOR was 4.812 (95% CI: 1.374–16.847). **Conclusions:** RBC transfusions increased the risk of ROP.

## 1. Introduction

### 1.1. Epidemiology

Retinopathy of prematurity (ROP) is a vascular proliferative disease that affects the developing retina of premature infants. The disease is caused by incomplete vascularization of the retina at birth. ROP is highly relevant from a public health perspective, as it remains a leading cause of childhood blindness worldwide, particularly in low- and middle-income countries in which the survival of premature infants has improved without being complemented by adequate preventive strategies targeting oxygen control and the development of ophthalmological screening and treatment programs [1,2,3]. The key to preventing ROP-related blindness lies in its early diagnosis and treatment [1,4].

Terry first reported ROP as retrolental fibroplasia in 1942. Oxygen plays a central role in ROP pathogenesis, which has been demonstrated by the dramatically reduced ROP incidence following oxygen restriction protocols. However, major advances in neonatal care leading to the survival of very-low-birth-weight (VLBW) and extremely premature infants (<28 weeks, <1000 g) have resulted in the re-emergence of severe ROP despite more cautious use of oxygen administration, generating the “second epidemic” in high-income countries [2,5].

### 1.2. Risk Factors

ROP is a multifactorial vascular proliferative disease that affects the retina of premature infants, involving numerous major and alternative risk factors [3,6].

The major factors for ROP development include low gestational age (GA), low birth weight (BW), and inadequate oxygen therapy, as reflected by prolonged or unregulated supplemental oxygen and poor oxygen monitoring.

The alternative risk factors for ROP development are summarized as systemic illness and poor postnatal growth, including respiratory and cardiopulmonary instability (prolonged mechanical ventilation and CPAP, bronchopulmonary dysplasia, respiratory distress syndrome, and surfactant use), neonatal morbidity (sepsis, necrotizing enterocolitis, intraventricular hemorrhage, patent ductus arteriosus, and metabolic acidosis), administration of blood products and hematologic conditions (multiple blood transfusions, erythropoietin use, and anemia), and use of certain medication (nitric oxide, dopamine, and sildenafil) [3,7].

Prematurity anemia is a moderate, context-dependent risk factor for ROP, acting primarily through worsening retinal hypoxia. It has been proven that multiple blood transfusions are associated with higher ROP rates [6,8,9].

### 1.3. Pathophysiology

The primary rationale of blood transfusions in preterm infants is the correction of anemia of prematurity with the aim of optimizing oxygen delivery to vital organs such as the brain, retina, myocardium, and gut [6,10].

After birth, physiological, self-limited anemia occurs. The trigger of this benign phenomenon is the sudden increase in oxygen availability after birth, leading to the down-regulation of erythropoietin (EPO) production and a subsequent temporary decrease in red blood cells. Accelerated red blood cell turnover also occurs, generated by the short lifespan of neonatal red blood cells (RBCs) (60–90 days) compared to adult RBCs (120 days), which contributes to falling hemoglobin (Hb) levels. Moreover, fetal Hb (HbF), which has a high affinity for oxygen, is progressively replaced by adult Hb (HbA) with lower affinity for oxygen, releasing it more efficiently. In term infants, physiological anemia is mild, late, asymptomatic, and requires no intervention. Anemia of prematurity is more severe than physiological newborn anemia due to several factors: more pronounced EPO suppression, shorter RBC lifespan (40–60 days, as compared to 90 days in term infants), lower Hb reserve at birth, rapid postnatal growth and plasma volume expansion, and iatrogenic blood loss, which is inherently associated with the need for medical care and a higher burden of systemic disease (chronic lung disease, late-onset sepsis, and bone disease) [6,9,11,12].

The low plasma levels of EPO in preterm infants are a consequence of the relative insensitivity of immature hepatic chemoreceptors to tissue hypoxia. The pathogenesis of anemia in premature infants is further compounded by relative deficiencies in micronutrients essential for erythropoiesis (iron, folate, vitamin E, and vitamin B12), in addition to insufficient maternal iron reserves, which may themselves be reduced due to maternal nutritional deficiency [6,12,13].

RBC transfusions may expose premature newborns to a higher risk of developing ROP [14], the severity of which is related to the timing and number of transfusions [15].

The aim of this study was to determine the risk of developing ROP in preterm infants with anemia in whom RBC transfusions were required.

### 1.4. Importance of the Study

Despite extensive research in this field, findings remain heterogenous and inconclusive. We therefore aimed for this study to serve as a step towards improving the ROP screening protocol in Romania with the goal of optimizing its sensitivity and specificity. In addition, a regional data gap exists (Romania/Eastern Europe), reflected by limited published data from the region, in addition to differences in NICU practices, oxygen protocols, and screening adherence. In this context, this study may provide added epidemiological value and enhance external validity.

## 2. Materials and Methods

### 2.1. Study Design and Participants

A retrospective cohort study was conducted on premature infants who met the following inclusion criteria: infants with gestational age (GA) ≤ 32 weeks and very low birth weight (VLBW) who were admitted to the Neonatology-Preterm Department of Emergency Pediatric Hospital Cluj-Napoca, Romania, during a two-year period (from 1 January 2023 to 31 December 2024).

The inclusion criteria correspond to the national ROP screening guidelines defined below. The exclusion criteria were as follows: (1) severe congenital malformations; (2) other ocular abnormalities that could affect ROP development or progression; (3) neonates that required exchange transfusion for hyperbilirubinemia.

### 2.2. Red Blood Cell Transfusion

Anemia impacts clinical status when the oxygen-carrying capacity drops below an adequate threshold to meet oxygen consumption demand. Consequently, symptomatic anemia does not occur at pre-defined Hb levels, but rather when there is an imbalance between oxygen delivery and consumption [16]. There is no universal guideline for RBC transfusion in anemia of prematurity. The attending physician determines the timing and volume of the transfusion based on a standard established at the level of each department, which includes clinical and laboratory criteria. Clinical criteria for RBC transfusion outline symptomatic anemia and are represented by respiratory distress syndrome, tachycardia, increasing oxygen/ventilation needs, poor feeding, and growth failure. Hb-based transfusion thresholds in very preterm infants (<32 weeks GA) vary according to clinical stability and respiratory support. In the first postnatal week, the Hb trigger for RBC transfusion varies between <10 g/dL in stable premature infants with no respiratory support and <13 g/dL in mechanically ventilated/critically ill infants. In the second postnatal week, the Hb trigger varies between <8.5 and 9 g/dL in stable, off-oxygen premature infants and <10 g/dL in the ventilated infants and after week 3, between <7 and 7.5 g/dL in stable, off-oxygen premature infants and <9 and 10 g/dL in ventilated infants. The typical transfusion practice at our facility consists of the transfusion of a dose of 10–20 mL/Kg packed RBC, at a rate of approximately 5 mL/kg/h, with the aim of increasing Hb by approximately 2 g/dL. Transfusion volumes and GA at the time of transfusion were not available for our study.

### 2.3. Definitions and Clinical Parameters

GA was calculated from the best obstetric estimate according to early prenatal ultrasound and obstetric examination. EPI is defined as GA less than 28 weeks. VPI is defined as GA 28 to less than 32 weeks. Moderate preterm infants were defined by GA 32 to less than 34 weeks.

Days on mechanical ventilation refer to the number of days of full-time exposure. Early- and late-onset sepsis were defined according to the standard in NICUs, namely, ≤72 h and >72 h, respectively. We defined sepsis according to clinical criteria, which included mandatory and supportive criteria represented by laboratory evidence of infection and microbiological confirmation when available [17]. Apnea of prematurity was defined as a cessation of breathing lasting ≥ 20 s or <20 s if accompanied by bradycardia, oxygen desaturation, and cyanosis/pallor that could not be explained by another identifiable cause [18].

Severe respiratory distress syndrome is caused by surfactant deficiency in preterm infants, leading to alveolar collapse and impaired gas exchange; as a consequence, they become hypoxic and require oxygen therapy or mechanical ventilation [3]. Bronchopulmonary dysplasia is diagnosed when the infant requires supplemental oxygen ≥ 28 postnatal days and at 36 weeks postmenstrual age (or discharge) if the infant still requires oxygen and/or respiratory support [3]. Patent ductus arteriosus is defined by the presence of a compatible heart murmur and a ductal right-to-left shunt visible on echocardiography [18]. Early blood transfusion was considered when RBC transfusion was required in the first 10 days of life [15].

### 2.4. Ophthalmological Screening and ROP Definition

At our facility, we perform ROP screening according to national guidelines, as follows: standardized ROP screening by means of indirect ophthalmoscopy is recommended for infants born ≤ 32 weeks of gestation (≤34 weeks of gestation before 2020) and/or with BW ≤ 1500 g independent of the need for supplemental oxygen, in addition to infants outside the above mentioned criteria in which risk factors for ROP were identified, including neonatal sepsis, necrotizing enterocolitis, intraventricular hemorrhage, hypoxia at birth, blood transfusions, treatment with dopamine for neonatal shock, and oxygen administration not in accordance with protocols. The period for screening initiation was 4 weeks postnatal age, but no earlier than 30/31 weeks of postmenstrual age (PMA) [19,20].

All patients were examined under pharmacological mydriasis and topical anesthesia by 3 experienced pediatric ophthalmologists (S.D.N., A.C.S., and M.C.H.) using indirect ophthalmoscopy with indentation and a 28-diopter lens. Depending on individual findings, examinations were performed weekly or every two weeks and continued until physiological vascularization reached zone 3 and/or pre-existing ROP showed marked and continuous regression. If the infant showed signs of ROP requiring treatment (zone I ROP at any stage with plus disease, zone I stage 3 without plus disease, or zone II stage 2 or 3 with plus disease), either intravitreal anti-VEGF injections (Bevacizumab) or laser photocoagulation was performed based on the ophthalmologist’s recommendation. ROP was defined according to the International Classification of ROP consensus statement [21]. Severe ROP was defined as stage 3 to stage 5 ROP or plus disease in zone I or II. ROP progression refers to progression from stage 1–2 to stage 3–4 [22].

### 2.5. Statistical Analysis

Bivariate analysis between infants with and without ROP was performed using the Mann–Whitney U test for non-parametric continuous variables, the independent samples t-test for parametric continuous variables, and the chi-square test for categorical variables. The chi-square test was used to assess the association between RBC transfusions and ROP.

Binary logistic regression was used to assess the association between RBC transfusions and ROP. Important covariates used in the final model included known risk factors for ROP such as gestational age, severe respiratory distress syndrome, and early- and late-onset sepsis.

The need for RBC transfusion was assessed through binary logistic regression using the covariates entered in blocks in a single step. In the regression model, we assessed the odds ratio, confidence interval, and Nagelkerke R^2^. Statistical analysis was performed using IBM SPSS Statistics version 29.0.

## 3. Results

### 3.1. Patient Characteristics

**Figure 1:**
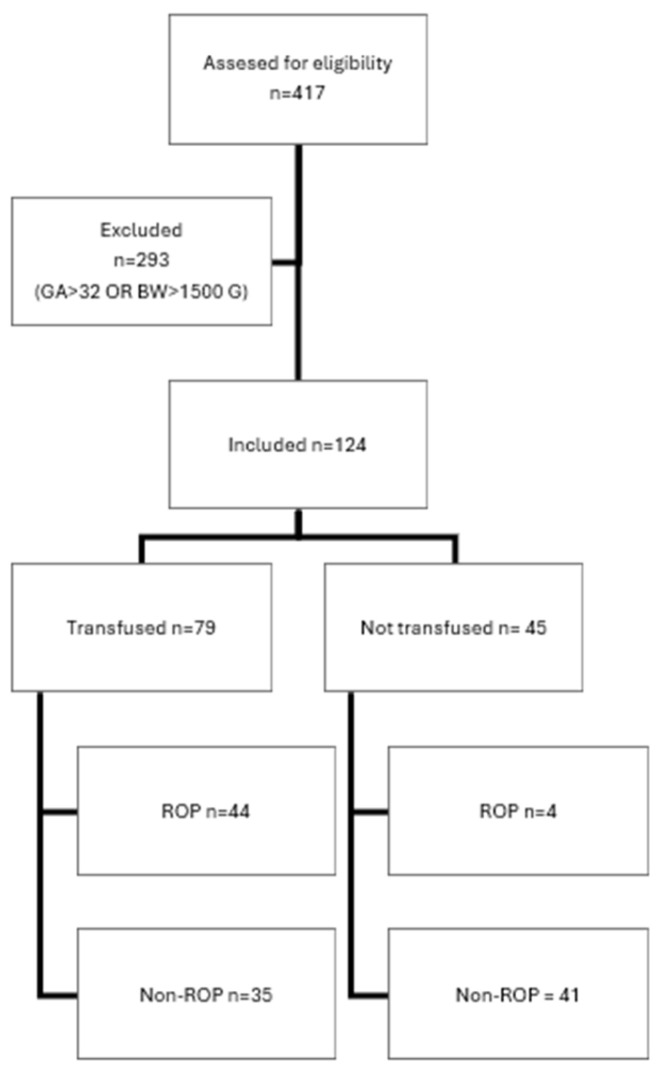
Flowchart of patients selection.

Patient selection was performed as illustrated in the flowchart below (Figure 1).

A total of 124 infants who met the inclusion criteria were included in the study. The median (IQR) GA was 28 (27–30) weeks, and the median (IQR) BW was 1100 (882.5–1365) grams. ROP developed in 48 preterm infants (incidence rate: 38.7%). The number of patients who required RBCs was 79 (63.7%). In Table 1, the results of the chi-square test are presented (*p* < 0.05).

### 3.2. Statistical Analysis

A chi-square test of independence was performed to evaluate the relationship between the development of ROP and the requirement for RBC transfusion. The relationship between these variables was significant, X^2^ (1, N = 124) = 26,475, *p* < 0.001. The infants who required RBC transfusions were more likely to develop ROP than those who did not. OR = 12.886, 95% CI [4.211–39.435].

Within the EPI group, all seven infants with GA < 25 weeks required RBC transfusions and developed ROP; they were thus excluded from the bivariate analysis.

The results of bivariate analysis are summarized in Table 2.

A binary logistic regression was conducted to determine the effects of RBC transfusion on the likelihood of developing ROP. The logistic regression model was statistically significant, X^2^ (5, N = 117) = 35.279, *p* < 0.001, indicating that the model distinguished between participants who will develop ROP and those who will not.

The model explained [Nagelkerke/Cox & Snell] (35.8%/26.0%) the variance in ROP development and classified 80.3% of cases correctly. Participants who required RBC transfusions were associated with an increased likelihood of developing ROP, OR = 4.812, 95% CI [1.374, 16.847], *p*= 0.014. The results of the binary logistic regression are highlighted in Table 3.

**Figure 2:**
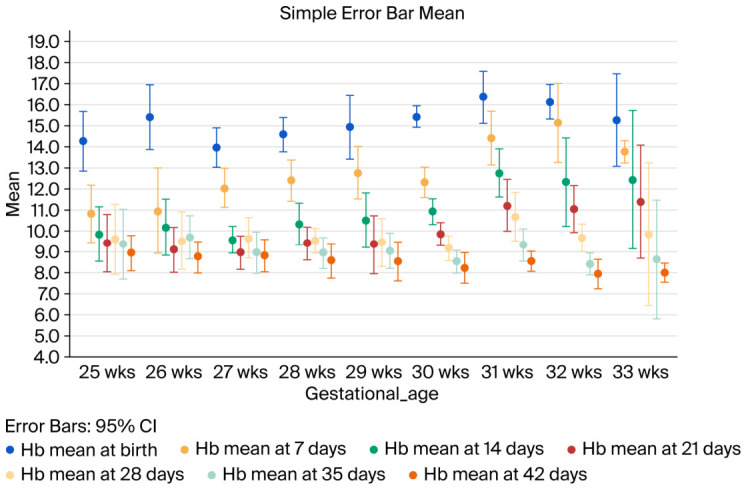
Hemoglobin values in relation to GA.

**Figure 3:**
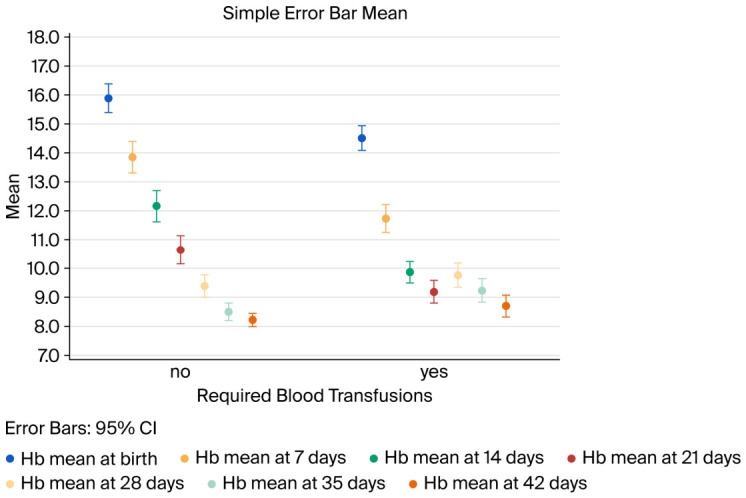
Hemoglobin values in relation to RBC transfusion requirements.

**Figure 4:**
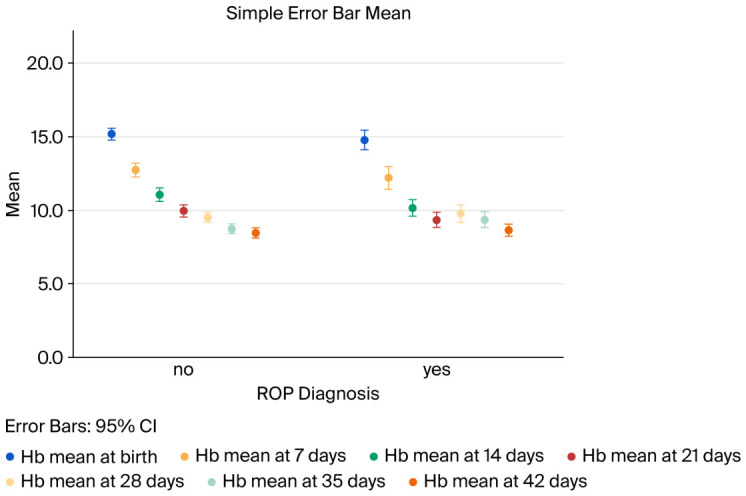
Hemoglobin values in relation to ROP.

We compared the mean hemoglobin value at birth, 7, 21, 28, 35, and 42 days in relation to GA, RBC transfusion requirement, and ROP diagnosis and found no statistically significant differences, as shown in Figure 2, Figure 3 and Figure 4.

## 4. Discussion

In our cohort, RBC transfusions were significantly associated with the development of ROP (OR 12.886, *p* < 0.001), indicating a markedly increased risk in transfused infants. This finding supports the hypothesis that transfusion exposure may play a significant role in ROP pathogenesis, potentially through enhanced oxygen delivery and oxidative stress-mediated retinal injury. Overall, RBC transfusion exposure appears to be a strong and modifiable risk factor for ROP.

The more premature the newborn, the earlier, more severe, and longer the duration of anemia. In EPIs, anemia typically develops after the first week of life and resolves by 40–44 weeks PMA. In the absence of universally accepted transfusion thresholds, the management of anemia in EPI is tailored according to various criteria: timing, PMA, cardiorespiratory support, and hemodynamic stability [8]. Balancing insufficient oxygen delivery against transfusion-related morbidity in a host with immature erythropoiesis and multiple comorbidities is a clinical dilemma that results in widely variable protocols between hospitals. The distinction between the role of anemia and RBC transfusions in the occurrence of ROP is incredibly difficult to establish, because the indication of transfusion is based on the presence of anemia, which in turn is involved in the pathogenesis of ROP through the hypoxic effect on the retina.

The pathogenesis of ROP consists of a biphasic process initiated by oxygen exposure in a preterm infant with an immature retina. The first phase (from birth to 30–32 weeks PMA) is called vaso-obliteration/vascular growth arrest, and it is driven by exposure to higher oxygen than intrauterine levels. Consequently, Vascular Endothelial Growth Factor (VEGF) and Insulin-like Growth Factor-1 (IGF-1) are suppressed, resulting in the arrest of physiologic angiogenesis, regression of existing immature vessels, and expansion of the avascular peripheral retina. The second phase (from 32 to 34 weeks PMA onward) is called hypoxia-driven pathologic neovascularization and consists of abnormal angiogenesis stimulated by retinal hypoxia in avascular areas. This phase is characterized by an imbalance between the increasing oxygen demands of a maturing retina and the lack of vascular development. Hypoxia triggers the overexpression of VEGF and other pro-angiogenic cytokines, leading to disorganized neovascular tufts, the growth of new vessels into the vitreous, and fibrovascular proliferation, which detaches the retina if untreated. Anemia worsens retinal hypoxia, thus representing an additional risk factor for ROP [23].

The role of anemia as a risk factor for ROP has been previously demonstrated [11,24,25,26,27].

In a retrospective cohort study performed on EPIs (GA < 28 weeks), Lundgren et al. evaluated the correlation between the risk of ROP and anemia and blood transfusions. Anemia was defined as Hb < 110 g/dL and it was considered severe at Hb < 80 g/dL and ROP was categorized as either warranting or not warranting treatment. The findings of this study demonstrated that in EPIs, the duration of anemia during the first week of life was associated with an increased risk of developing ROP, warranting treatment [24].

Pheng et al. compared, retrospectively for the first six weeks of life, the mean weekly Hb levels between premature infants with and without ROP. Their findings demonstrated that in premature infants with GA < 32 weeks, Hb levels were significantly lower in the ROP than in the non-ROP group. The difference was significant only during the first week of life, neither at birth nor from 2 to 6 weeks after birth [25].

In a retrospective study, Pai et al. investigated the correlation between the incidence of ROP and risk factors such as Hb levels in premature infants born before 34 weeks of GA. Their findings demonstrated that Hb level was significantly associated with ROP incidence and severity [26].

Maeda et al. evaluated the association between RBC parameters and the need for ROP treatment. The authors proved that infants with GA < 30 weeks who at birth presented mean corpuscular volume (MCV) > 117.3 fL, Hb < 9.9 g/dL, and hematocrit (Hct) < 31.0% had a significantly higher risk of developing ROP requiring treatment [27].

In a study performed by Tandon et al., anemia was identified as a significant risk factor for ROP and the statistically significant mean Hb values were set in correlation with the ROP stage, as follows: 10.41 g/dL for stage 1, 10.56 g/dL for stage 2, 9.47 g/dL for stage 3, 9.3 g/dL for stage 4, and 12.15 g/dL for the matured retina [11].

In numerous studies, RBC transfusions have been associated with the development of ROP such that the more RBC transfusions performed, the greater the risk of ROP [28,29,30]. This situation creates a clinical dilemma as to whether to maintain low oxygen levels to prevent ROP or to maintain high oxygen levels to prevent complications such as cerebral palsy. As such, further research is needed regarding the indications for oxygen therapy and blood transfusions.

Observational studies such as the one conducted by Ghirardello et al. demonstrated that RBC transfusion was an independent risk factor for treatment-requiring ROP, even after adjustment for GA and illness severity. The association was the strongest in EPIs and proportional to the increasing number of transfusions. The findings of the afore-mentioned study demonstrated that three or more transfusions increased the risk of ROP by 4.88 times [28].

Hengartner T et al. demonstrated in their study that infants with ≥stage 2 ROP, in a defined geographical area, received larger volume transfusions and required earlier treatment [29].

In their study, Uberos et al. demonstrated that the number of RBC transfusions has a more significant impact on the risk of developing ROP and severe ROP than early RBC transfusions (within the first 7 days of life). They observed that the administration of the first RBC transfusion during the vaso-obliterative phase of ROP (<32 weeks of corrected GA) is significantly associated with an increased risk of ROP [30].

In our study, the adjusted logistic regressions demonstrated that RBC transfusions were a risk factor for developing ROP. We were unable to retrieve the volume of each RBC transfusion from the medical records of our patients. Therefore, we could not determine the dose-dependent effects of RBC transfusions on ROP. However, through multivariate statistical analysis, we were able to separate the independent effects of anemia, transfusions, and disease severity.

RBC transfusions lead to ROP through multiple pathways acting on immature retinal vasculature during a vulnerable period. The key role is attributed to the replacement of HbF with HbA [31], which has a lower affinity for oxygen and thus exposes the immature retina to high oxygen levels with subsequent oxidative stress, downregulation of VEGF expression, and inflammatory endothelial cell dysfunction. All of these factors result in retinal hypoxia, which triggers VEGF expression, consequently leading to ROP development through the proliferation of abnormal vessels in the retina and vitreous [6].

There is a delicate balance between Hb level and ROP pathogenesis. Both low Hb (anemia) and high Hb achieved through transfusions can increase ROP risk, but through different mechanisms. Low Hb leads to a decrease in oxygen delivery to the retina and subsequent retinal hypoxia, which triggers an increase in endogenous EPO and VEGF expression in the avascular retina. This process promotes pathological neovascularization of the retina and vitreous (phase 2 ROP). High Hb achieved through RBC transfusions increases ROP risk through the replacement of HbF with HbA, which releases oxygen more rapidly, leading to relative retinal hyperoxia with subsequent suppression of VEGF and IGF-1 and halting or regression of vessel growth. This process drives phase 1 ROP (vascular obliteration) [32]. This delicate balance requires consideration of the simultaneous effects of low oxygenation levels (death, cerebral palsy, patent ductus arteriosus, pulmonary vascular resistance, and apnea) and hyperoxygenation (ROP and chronic lung disease) [33].

Multiple RBC transfusions may further contribute to retinal damage by promoting the accumulation of free iron and subsequently increasing the production of free hydroxyl radicals in the retinal tissue [34]. In our study, all six patients who required more than three blood transfusions developed ROP requiring treatment; however, these patients also had a GA < 25 weeks.

Preventing anemia and reducing the risks associated with RBC transfusions are challenging problems in neonatology. Various methods have been proposed: delayed cord clamping (≥30–60 s after birth), minimization of iatrogenic blood loss, using restrictive thresholds for RBC transfusion, iron supplementation, and erythropoiesis-stimulating agents [35].

In a prospective cohort study describing neonatal transfusion practices across Europe, the authors found differences in thresholds, volumes, durations, and infusion rates between countries. Further research is required to address these variations and define optimal practice [36].

To reduce the impact of anemia and RBC transfusions on the pathogenesis of ROP, umbilical cord blood (UCB) transfusion has been proposed as an alternative strategy. The rationale for collecting autologous blood at birth and transfusing it later in the premature infant is based on treating anemia while reducing exposure to adult RBCs and preserving HbF, which has higher oxygen affinity, reduced oxygen toxicity risk, and is more stable in an environment of oxidative stress. UCB also contains growth factors and progenitor cells that may have a favorable impact on ROP. A study protocol of UCB transfusion, with ROP incidence as the primary outcome, was proposed by Torrejon-Rodrigues L. et al. [37]. UCB transfusion remains an experimental concept that is practically limited by the insufficient volume in EPIs, storage and sterility issues, and incompatibility with current routine NICU practice.

In our observational study, we report the significant association between RBC transfusions and ROP, similar to most observational studies, suggesting a causal role. However, more critically ill and more premature infants require transfusions, with them also having a higher intrinsic risk of ROP. In this context, transfusions appear to be a marker, not a cause, and their contribution to the occurrence of ROP may be overestimated. The retrospective nature of the study did not allow us to establish the exact timing of RBC transfusions, with the knowledge that if transfusions occur after retinal vascular dysregulation begins, causality weakens. Nevertheless, a direct biological contribution cannot be excluded, given the potential effects of adult hemoglobin, oxidative stress, and altered oxygen delivery on the developing retina. Therefore, RBC transfusions can be attributed to the role of a composite risk indicator, which integrates both the severity of systemic illness and the direct effect on retinal vascular development.

Our study has several limitations that must be addressed. First, the retrospective, single-center design carries a high risk of selection bias and missing data and limits the generalizability to other settings. Moreover, only association, but not causality, can be established. Second, the small sample size reduces statistical power and increases the risk of type II and type I errors. Third, critical transfusion data are absent: timing (early vs. late), volume per transfusion, and transfusion thresholds. Fourth, the patients who received a greater number of RBC transfusions were critically ill and smaller, carrying a higher risk of ROP development; however, using the regression model, we adjusted for the illness severity of the infants. Fifth, our study lacks cofactors to perform a full logistic regression. Thus, important risk factors for ROP development, such as oxygen exposure (dose/duration), mechanical ventilation duration, nutritional status, and iron therapy/EPO use, were not available for analysis. Thus, the absence of these key exposure variables limits causal inference and suggests that reported associations should be interpreted as hypothesis-generating rather than definitive.

## 5. Conclusions

Based on the results of our study, RBC transfusions increased the risk of ROP development. Further, larger, multicenter prospective studies are required to establish the causality, not merely the association, between RBC transfusions and ROP, with a focus on volume per transfusion and transfusion thresholds. We recommend that for VPIs and EPIs with small GA, low BW, and a high risk of sepsis, the indications of RBC transfusions be strictly controlled in terms of number and volume, to reduce adverse events.

## Figures and Tables

**Table 1:** Cross-tabulation of patients who developed ROP and required RBC transfusion.

	ROP	Without ROP	Total
Transfused	44	35	79
Not transfused	4	41	45
Total	48	76	124

**Table 2:** Bivariate analysis of patients with ROP and those without.

Risk Factors	ROP N = 41	Without ROP N = 76	*p* Value
Gestational age (weeks), median (IQR) ^1^	27 (26–29)	30 (28–30)	<0.001
Birth weight (grams), mean ± SD ^2^	923.29 ± 43.655	1222.24 ± 29.145	<0.001
Female, *n* = 56 ^3^	22	34	0.467
C-section delivery, *n* = 84 ^3^	29	55	1
Severe respiratory distress syndrome, *n* = 48 ^3^	27	21	<0.001
Patent ductus arteriosus, *n* = 78 ^3^	28	50	0.945
Early-onset sepsis, *n*= 69 ^3^	29	40	0.089
Late-onset sepsis, *n* = 37 ^3^	21	16	0.002
Bronchopulmonary dysplasia, *n* = 27 ^3^	19	8	<0.001
Number of RBC transfusions performed, median (IQR) ^1^	2 (1–4)	0 (0–2)	<0.001
Required RBC transfusion, *n* = 72 ^3^	37	35	<0.001
Required more than 3 RBC transfusions, *n* = 16 ^3^	13	3	<0.001
Required early RBC transfusion, *n* = 19 ^3^	12	7	0.011

^1^ Mann–Whitney U test; ^2^ Independent sample *t*-test; ^3^ Chi-square test.

**Table 3:** Logistic regression model for the need for RBC transfusion and ROP development.

							95% C.I. for Exp(B) (OR)	
	B	S.E.	Wald	df	Sig.	Exp(B)	Lower	Upper
Need for RBC transfusion	1.571	0.639	6.038	1	0.014	4.812	1.374	16.847
Severe respiratory distress syndrome	0.616	0.548	1.266	1	0.261	1.852	0.633	5.417
Early-onset sepsis	−0.058	0.516	0.013	1	0.910	0.943	0.343	2.594
Late-onset sepsis	0.549	0.501	1.198	1	0.274	1.731	0.648	4.623
Gestational age	−0.234	0.165	2.022	1	0.155	0.791	0.573	1.093
Constant	4.453	5.021	0.787	1	0.375	85.862		

B = log-odds coefficient; Exp(B) = Odds Ratio (OR); 95% C.I. = 95% Confidence Interval for Exp(B). If the C.I. includes 1.0, the effect is not statistically significant.

## Data Availability

The original data presented in the study are openly available on FigShare at https://doi.org/10.6084/m9.figshare.32083362 (accessed on 16 June 2026).

## Abbreviations

The following abbreviations are used in this manuscript:

ROP	Retinopathy of prematurity
RBCs	Packed red blood cells
VLBW	Very low birth weight
GA	Gestational age
Hb	Hemoglobin
HbF	Fetal Hemoglobin
HbA	Adult Hemoglobin
EPO	Erythropoietin
EPI	Extremely preterm infants
VPI	Very preterm infants
PMA	Postmenstrual age
MCV	Mean corpuscular volume
Hct	Hematocrit
RDW	Red cell distribution
DOL	Day of life
UCB	Umbilical cord blood
